# Perovskite Quantum Dots for Emerging Displays: Recent Progress and Perspectives

**DOI:** 10.3390/nano12132243

**Published:** 2022-06-29

**Authors:** Xinxin Ren, Xiang Zhang, Hongxing Xie, Junhu Cai, Chenhui Wang, Enguo Chen, Sheng Xu, Yun Ye, Jie Sun, Qun Yan, Tailiang Guo

**Affiliations:** 1 National & Local United Engineering Laboratory of Flat Panel Display Technology, College of Physics and Information Engineering, Fuzhou University, Fuzhou 350108, China; rxx7321@163.com (X.R.); 111901140@fzu.edu.cn (X.Z.); xiehongxing1992@163.com (H.X.); 15711526574@163.com (J.C.); ahbcxc@163.com (C.W.); xusheng06090@163.com (S.X.); yeyun07@fzu.edu.cn (Y.Y.); jie.sun@fzu.edu.cn (J.S.); qunfyan@gmail.com (Q.Y.); gtl@fzu.edu.cn (T.G.); 2 Fujian Science & Technology Innovation Laboratory for Optoelectronic Information of China, 2 Xueyuan Road, Fuzhou 350108, China

**Keywords:** perovskite, quantum dots, synthesis, photoluminescent displays, electroluminescent displays

## Abstract

The excellent luminescence properties of perovskite quantum dots (PQDs), including wide excitation wavelength range, adjustable emission wavelength, narrow full width at half maximum (FWHM), and high photoluminescence quantum yield (PLQY), highly match the application requirements in emerging displays. Starting from the fundamental structure and the related optical properties, this paper first introduces the existing synthesis approaches of PQDs that have been and will potentially be used for display devices, and then summarizes the stability improving approaches with high retention of PQDs’ optical performance. Based on the above, the recent research progress of PQDs in displays is further elaborated. For photoluminescent display applications, the PQDs can be embedded in the backlighting device or color filter for liquid crystal displays (LCD), or they may function as the color conversion layer for blue organic light-emitting diodes (OLED) and blue micro-scale light-emitting diodes (μLED). In terms of next-generation electroluminescent displays, notable progress in perovskite quantum-dot light emitting diodes (PeQLED) has been achieved within the past decade, especially the maximum external quantum efficiency (EQE). To conclude, the key directions for future PQD development are summarized for promising prospects and widespread applications in display fields.

## 1. Introduction

Photoluminescence (PL) and electroluminescence (EL) are two fundamental excitation modes for current self-emissive displays. EL displays are commonly driven by a given electron current [1,2], whereas the PL type [3] is enabled by down-conversion luminescence materials [4], such as phosphors [5] and quantum dots (QDs) [6,7]. These luminescence materials can be flexibly integrated into light sources, backlight components, functional films, or display panels. They, to a great extent, determine the display performance, and thus need to provide high luminescence efficiency, high color purity, and good stability. The well-known YAG:Ce^3+^ was initially used as the basic material of display phosphor for backlighting devices [8]. However, the wide emission width of this phosphor provides a limited color gamut of merely ~72% according to the National Television System Committee (NTSC) standard [9]. This can be markedly improved by narrow-band red and green-emitting phosphors [10,11,12], such as the popular green phosphor Beta SiAlON:Eu^2+^ (~525 nm/~50 nm), red phosphor K2SiF6:Mn^4+^ (~630 nm/~5 nm), etc. Even so, they are still not qualified for next-generation high-resolution and wide-color-gamut displays.

**Figure 1:**
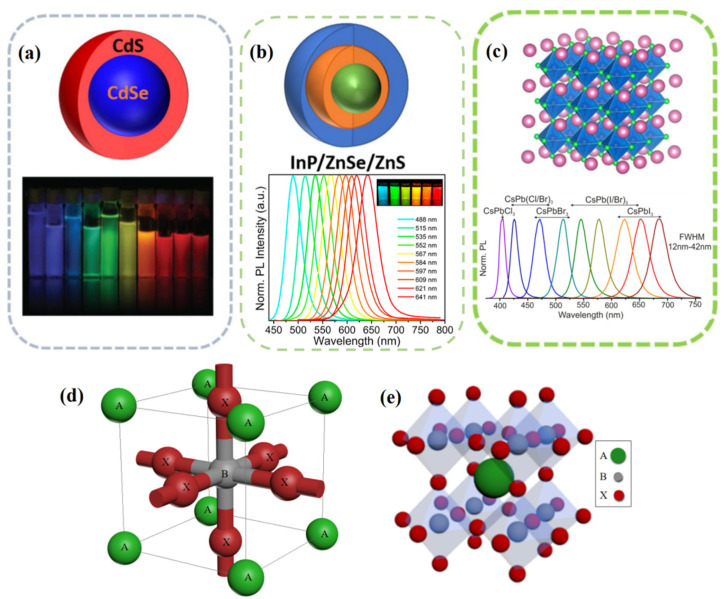
(**a**) CdSe-based QDs. (**b**) InP-based QDs. (**c**) PQDs. Adapted with permission from [22]. Copyright 2020, American Chemical Society. (**d**) The octahedral unit and (**e**) network structure of perovskite ABX_3_.

In display fields, a new luminescence material is gradually replacing phosphors due to its narrow emission width, saturated color, and tunable emission [13,14]. This material is named QDs because of its quantum confinement effect and nanoscale dimension [15]. There are roughly three kinds of QD materials for display application: II–VI semiconductor QDs, III–V semiconductor QDs, including perovskite QDs (PQDs), of which the corresponding representatives are shown in Figure 1a–c. Cadmium chalcogenide CdSe-based QDs with a core-shell structure have been successfully commercialized in displays because of their high PL quantum yield (PLQY, ~100%), color quality (FWHM, ~20 nm), and good stability [16,17]. However, the constituent toxic element, Cd, has raised environmental and health concerns. For this reason, InP-based QDs with comparable efficiency and slightly lower color purity have been developed as an alternative [18,19]. To guarantee their performance, the typical InP QD structure is required, which consists of an InP/ZnSe/ZnS core–buffer shell–outer shell structure. This causes the complex and time-consuming synthesis process. In addition, raw materials, especially phosphorus precursor, are expensive.

As a novel kind of QDs, PQDs has gained increasing attention. Compared with the above-mentioned QDs, PQDs exhibit inherent superiorities, including excellent luminescence performance, ease of synthesis, and conveniently tunable emission [20]. These characteristics originate from their unique structural attributes and make them a promising candidate in current and future displays. However, their intrinsic drawbacks also deserve close attention, especially instability and large-scale synthesis [21]. These unresolved issues hinder the breakthroughs in the display industry.

Therefore, this review focuses on the critical demands in display fields, and provides a comprehensive discussion to bridge the gap between fundamental knowledge and display applications. The key content of this review can be divided into three parts. Firstly, the basic synthesis methods of PQDs are concluded, which are deeply discussed from the perspective of PQD’s structure and performance. Secondly, we summarize and clarify the strategies for the stability improvement of PQDs in display applications, which is the most difficult issue for display applications. Finally, the latest research progress of PQDs in display fields is described, and the future development direction of PQDs and their display applications are prospected. Beyond other reviews on PQDs, this review provides inspiration for PQDs optimization in display fields, and promotes their application in current and future displays.

## 2. Fundamental Structure and Optical Properties of PQDs

### 2.1. Fundamental Structure of PQDs

Halide perovskites have a general formula of ABX_3_, where A and B are, respectively, monovalent and divalent cations, and X is a monovalent halide (Cl, Br, I) anion. The basic structural unit of metal halide perovskites is shown in Figure 1d,e, where B-site cations, usually Pb and Sn, will form inorganic octahedra with the six surrounding halide ions. However, when the B site is a mixed cation such as B ^+^ and B’ ^3+^, the whole structure will form a double-calcite structure with larger crystals. Cubic-phase perovskite (the most regular perovskite) has a corner-sharing structure, which means that the cation at site A is shared by eight neighboring cells, with the location of A at the apex of the cell [23].

The common metal halide perovskites can be further classified into either organic–inorganic (hybrid perovskite quantum dots, HPQDs) or all-inorganic perovskite quantum dots (IPQDs), depending on whether the A cation is an organic molecule such as methylammonium (CH_3_NH_3_^+^) and formamidinium (FA^+^), or an inorganic cation (commonly Cs^+^). The optical and electronic properties of perovskites can be tunable by varying the composition of constituted halide ions and the size of the cations [24,25]. In addition, the dimensionality of perovskites can also be used to tune their optical properties, similar to conventional metal chalcogenide semiconductors [26,27]. Moreover, the reported dimensionality of perovskite can range from the 3D to 0D. Compared with the high-dimensional one, the low-dimensional perovskite nanocrystals (NCs) exhibit very high PLQY partly due to their defect tolerance [25,26,27,28,29,30], high exciton binding energy [31,32,33], high optical absorption coefficient [34,35], and tunable carrier diffusion length [36,37,38].

### 2.2. Optical Properties of PQDs

After PQDs are excited by external energy, the electrons in the valence band leap into the conduction band, and therefore holes are generated in the valence band. The three types of luminescence are as follows [39]. (1) The electrons return to the valence band and recombine with the holes to emit photons. (2) Electrons are trapped by a defect energy band to emit light. (3) Electrons are trapped by a doped energy level and then emit light.

Four basic optical parameters, including emission stability [40], luminous intensity [40,41,42], color diversity [43,44], and color purity [45], can be used to characterize the luminescence properties of PQDs. It is remarkable that these basic characteristics also determine their application feasibility in display devices. Among them, emission stability is related to the crystal lattice of PQD materials, and luminous intensity is mainly determined by PLQY. Color diversity can be regulated by changing the PQDs’ particle size, composition, and type of ionic elements, while color purity is associated with the FWHM of the emission spectrum. 

Compared with organic fluorescent dyes and rare-earth-doped phosphors, PQDs show excellent optical properties in the following four aspects: wide excitation wavelength range [22,40], high PLQY [40,41,42], adjustable emission wavelength [43,44], and narrow emission FWHM [45].

**(1). Wide excitation wavelength range**. The excitation spectra of both organic fluorescent dyes and rare-earth-doped phosphors are relatively narrow and may require the use of excitation sources in specific bands to obtain a desired emission spectrum. By contrast, the excitation spectrum of PQDs is continuous and can be excited by arbitrary light higher than the bandgap energy. Therefore, the same excitation light source can simultaneously excite PQDs with different band gaps, resulting in different fluorescent colors.

**(2). High PLQY**. PQDs with a high molar absorption coefficient have excitation overlap regions so that they can absorb large amounts of excitation light for light conversion. In addition, the defect energy levels caused by internal or surface defects in PQDs can be eliminated by optimizing synthesis methods, modified ligands, and coating, so as to obtain high quantum yield by radiation recombination.

**(3). Adjustable emission wavelength**. Due to the quantum confinement effect [46], the energy band of the semiconductor is split into discrete energy levels, resulting in different sizes of PQDs having different band gaps. In other words, by regulating the particle size, the light-emitting color of PQDs can be easily tuned to the required wavelength range for various applications. Different from other QDs, the luminescence color of PQDs can also be changed by controlling the components of the halogen anions, which has the potential to cover the entire visible spectrum.

**(4). Narrow emission FWHM**. For PQDs, the relaxation rate of electrons and holes in the band is much higher than the composite rate of thermal excitons. It is hard to have recombination between high-level electrons and holes. Thus, the luminescence spectra of monodispersed PQDs are basically symmetrical. The FWHM is comparable to the low-energy edge of the first exciton absorption peak, and the luminescence peak energy is slightly lower than the first exciton absorption peak.

**Figure 2:**
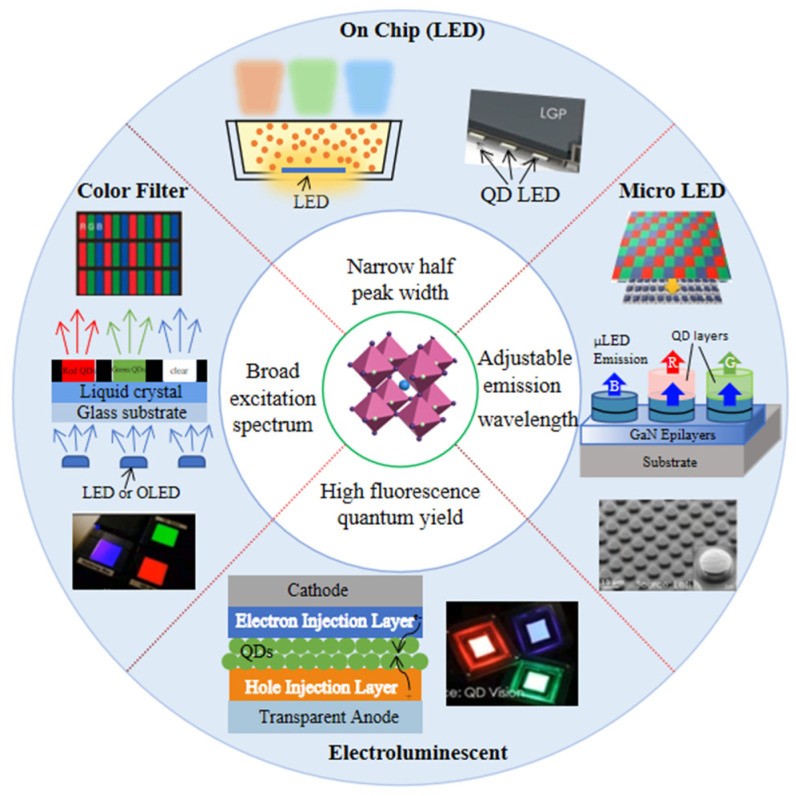
Schematic diagram of the research direction of PQD displays.

Based on the above excellent optical properties, PQDs show outstanding performance to better meet the need in emerging display, as shown in Figure 2.

## 3. Synthesis Methods of PQDs

**Figure 3:**
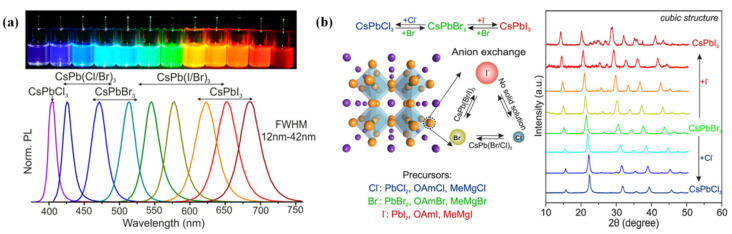
(**a**) Colloidal perovskite CsPbX_3_ NCs (X = Cl, Br, I) covering the whole visible spectral region with narrow and bright emission. Adapted with Open Access from [26]. Copyright 2015, American Chemical Society. (**b**) The anion exchange diagram and X-ray diffraction diagram of PQDs. Adapted with permission from [48]. Copyright 2015, American Chemical Society.

The synthesis of CsPbX_3_ PQDs showing bright emission and a wide color gamut was first reported by Loredana et al. in 2015, and it is widely known as the hot injection method [47]. The Cs-oleate precursor was prepared in advance, and then injected into a PbX_2_ (X = Cl, Br, I) solution dissolved in oleic acid (OA), oleylamine (OAm), and octadecene (ODE) at high temperature and in a nitrogen atmosphere. After a few seconds, the temperature of the reaction system was quickly dropped to room temperature and the PQDs could be obtained via centrifugation. By using this method, the cubic CsPbX_3_ QDs with a PLQY of 50~90% and an FWHM of 12~42 nm were successfully synthesized (Figure 3a), which paved a new way for the development of perovskite. The hot injection method introduces OA, and OAm ligands, providing potential for subsequent studies of ligand modification. In addition, this method facilitates the introduction of ions into perovskite lattice, laying the foundation for the study of ion doping. In the same year, Nedelcu et al. [48] prepared IPQDs by introducing different halogen elements into CsPbX_3_ QDs for an anion exchange reaction (Figure 3b), and finally realized the full spectral luminescence (410~700 nm) with the PLQY of 20~80%.

**Figure 4:**
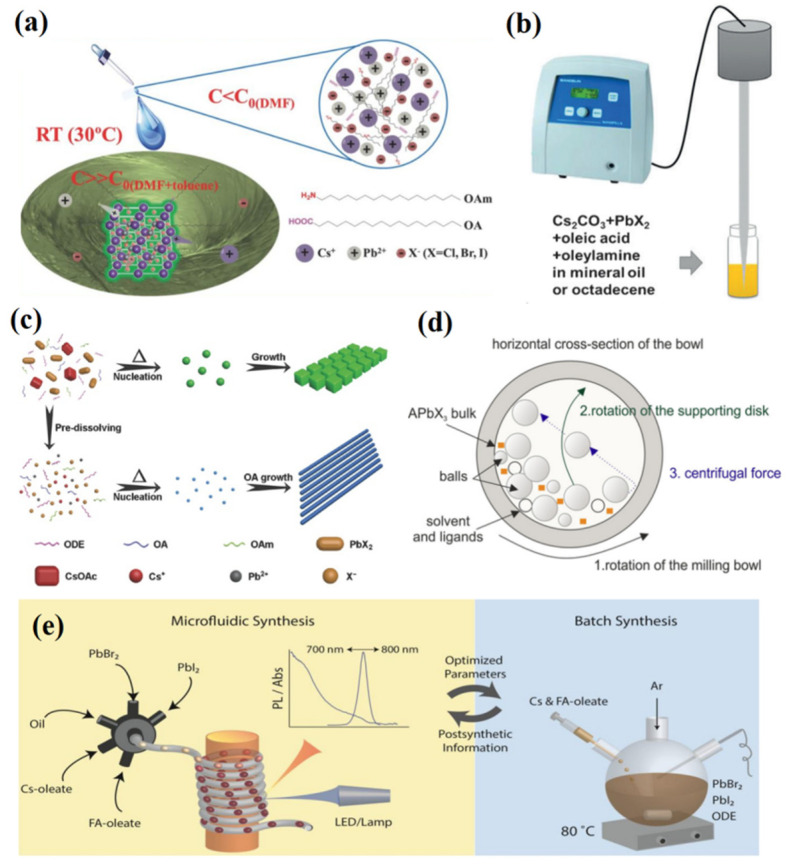
(**a**) Room-temperature precipitation method. Reproduced with permission from [49]. Copyright 2016, Wiley. (**b**) Ultrasonic method. Reproduced with permission from [50]. Copyright 2016, Wiley. (**c**) Solvothermal method. Reproduced with permission from [52]. Copyright 2017, Wiley. (**d**) Wet ball milling method. Adapted with Open Access from [54]. Copyright 2018, American Chemical Society. (**e**) Automatic microfluidic method. Adapted with Open Access from [58]. Copyright 2018, American Chemical Society.

Due to strict reaction conditions and a complex process, the hot injection method is still difficult for mass production at present. In 2016, the room-temperature reprecipitation method was proposed based on the differences in the solubility of ions in different solvents [49]. OAm and OA as surface ligands and PbX_2_ and CsX as ion sources were dissolved in dimethylformamide (DMF) at room temperature to serve as precursors. The precursor at a supersaturated state was injected into the toluene solution and a large number of perovskite crystals were precipitated (Figure 4a). The resulting perovskite had excellent optical properties, with a PLQY of 80%, 95%, and 70% and a FWHM of 35 nm, 20 nm, and 18 nm for red, green, and blue, respectively. In this method, ligands function to passivate the QDs’ surface to reduce surface defects and inhibit nonradiative recombination to improve the luminescence performance and lifetime. In addition, it is simple to operate without high temperature and an inert gas environment, and it is less affected by the environment. Therefore, it does have high repeatability compared with the hot injection method. In the same year, Tong et al. [50] described a universal nonpolar solvent ultrasound method which mixed precursors of cesium and lead halide with the end-sealing ligand (OAm and OA) in ODE followed by sonication for 10 min (Figure 4b). The PLQY of the prepared red, green, and blue perovskites, respectively, reached 90%, 92%, and 10%, and the synthesized CsPbBr_3_ was highly monodisperse. This simple, fast, and ligand-modifiable method is expected to achieve commercial production of perovskite.

In 2017, the microwave-assisted synthesis of CsPbX_3_ NCs with different morphologies was first reported by Pan et al. [51]. Cesium acetate, lead halide (PbX_2_, X = Cl, Br, I or their mixtures), a certain amount of trioctylphosphine oxide (TOPO), OA, OAm, and ODE were mixed in a microwave quartz tube and then put into a microwave reactor. Nanoplates and nanocubes were obtained at low and high reaction temperature, respectively, while nanorods could be formed by pre-dissolving precursors. This method provided uniform particle size distribution, simple operation, no inert gas, and less environmental impact. Compared with the hot injection method, it has a high repeatability and no other pretreatment. In the same year, the solvothermal method was proposed for the synthesis of IPQDs [52]. Cs_2_CO_3_ and PbX_2_, used as precursors, were mixed with OA, OAm, and ODE in the autoclave and maintained at 160 ℃ for a while (Figure 4c). CsPbX_3_ QDs and ultrathin nanowires with uniform cubic phase were successfully prepared with the PLQY reaching 80%. Although this simple preparation method could obtain high-quality IPQDs with controllable morphology, the uncontrollable system temperature made it rarely used in doping strategies and ligand modification.

The mechanochemical synthesis method was first proposed in 2017 [53]. Solid PbBr_2_, ABr, and capping ligands were mixed and ground at room temperature for a while. Square and rectangular (CsPbBr_3_), spherical (MAPbBr_3_), and parallelogram (FAPbBr_3_) nanoparticles (NPs) were prepared through this solid-phase method. Although its PLQY of 13% was significantly lower than in traditional liquid-phase method, it still showed certain good characteristics such as high yield, simplicity, and fast synthesis process. Due to the solid reaction system, the ligand modification strategy was hardly applied. For this reason, the wet ball milling method for preparing colloidal nanocrystals was further proposed by Kovalenko et al. in 2018 [54], which was composed of APbBr_3_ mixed with solvent and oil-based ammonium bromide ligand. Figure 4d illustrates the working principle of the wet ball milling method. In 2019, Palazon et al. [55] revealed the process mechanism, dynamics, and possible side effects of dry ball milling. The changes of mechanochemical synthesis with different time variations were studied in detail, and it was found that the drying and rapid (5 min) process affected the excellent phase purity of IPQDs. 

In 2018, Guo et al. [56] synthesized CsPbX_3_ microcrystals using chemical vapor deposition (CVD) at room temperature. The working process was summarized as follows. PbX_2_ and CsX (X = Cl, Br, I) were mixed in a reaction chamber. The substrate could be made of sapphire, SiO_2_, or Si. The products CsPbI_3_, CsPbBr_3_, and CsPbCl_3_ were grown at 580 °C, 620 °C, and 620 °C, respectively, with argon at the rate of 100 mL/min and the growth time of 30 min. The fluorescence lifetime was 59.7 ns (CsPbI_3_), 36.9 ns (CsPbBr_3_), and 3.5 ns (CsPbCl_3_), respectively. By using this method, the white-light-emitting chips could be successfully prepared on substrates. Though the large size (μm) and high precision of the experimental equipment still limit its application, it does show a certain potential in display backlights due to excellent performance of the crystal product.

In recent years, the synthesis of various kinds of nanocrystals with good homogeneity using a fully automated microfluidic platform has become a hot research topic [57,58]. This microfluidic platform allows the parameters of the synthesized nanocrystals to be varied by changing the precursor molar ratio (e.g., Cs/Pb, FA/Pb, Cs/FA, and Br/I), growth time (determined by flow rate and tube length), and temperature in a systematic and independent way. Droplets are generated by adjusting the flow rates of the carrier phase (50~200 μL/min) and that of the dispersed phase (1.2~50 μL/min) (Figure 4e left), and each droplet can be viewed as a small hot-injection reaction system (Figure 4e right). In 2018, Lignos et al. [58] further explored the synthesis of colloidal QDs in the near infrared using a microfluidic platform. The synthesis results showed that untreated colloidal QDs had an emission FWHM within 45~65 nm. The NCs could further narrow the PL FWHM to 40 nm after a series of post-treatments (e.g., isolation, size selection, and purification), while the synchrotron X-ray scattering clearly showed a cubic structure of Cs_x_FA_1__−x_Pb(Br_1__−y_I_y_)_3_ NCs. Finally, the electroluminescent devices prepared by this colloidal QDs obtained a 5.9% EQE.

To sum up, efficient, convenient, and low-cost synthesis methods have been proposed for PQDs, which lay a foundation for its potential applications in displays. Table 1 summarizes the characteristics, advantages, and disadvantages of the existing perovskite synthesis methods, with references attached.

## 4. Performance Improvement of PQDs

Although PQDs show high PLQY, low Auger recombination loss, and large exciton binding energy, the poor humidity resistance and thermal stability of PQDs greatly limit their practical application in the display field. Effective stabilization measures inevitably result in a decline in optical performance. This section focuses on the strategies to improve the stability and optical performance of PQDs.

### 4.1. Ion Doping of PQDs

An ion doping strategy has become an important way to improve the oxygen/moisture resistance and luminescence properties of PQDs, which initially came from the doping strategy of semiconductor QDs. ABX_3_ has three different lattice positions, and different lattice positions have different effects on the material. Taking CsPbBr_3_ as an example, Cs has little effect on its electronic structure. However, the 4p orbital of Br and the 6p orbital of Pb contribute greatly to the valence band and conduction band of the crystal, respectively. In addition, the excitation and recombination of electrons and holes are carried out in an octahedron [58]. Therefore, inorganic octahedrals are very important for the luminescence of PQDs, and different ionic doping will have different effects on the properties of PQDs.

The A-site has a great influence on the structure and stability of PQDs. Considering the valence distribution of perovskite lattice, monovalent cations (such as BA^+^ [59]) are mostly used as A-site doped ions. Because of the strong oxidation resistance, alkali metals (Na^+^ [60], K^+^ [61,62], Rb^+^ [63], etc.) are regarded as ideal doping ions at the A-site. In 2018, Liu et al. [61] doped CsPbCl_3_ with K^+^. The introduction of K^+^ reduced the defects of perovskite and narrowed the FWHM of the emission peak. With the increase of doping concentration, the PLQY increased from 3.2% to 7.2%, and the average lifetime was also improved. In the same year, Huang et al. [62] adopted Rb^+^ doping and found that when the proportion of Rb^+^ was close to 75%, the PLQY of PQDs could be effectively improved. Among them, the PLQY of blue PQDs changed most significantly, from 45% to 86%. Rb^+^ doping also effectively improved the UV light stability and thermal stability of perovskite.

**Figure 5:**
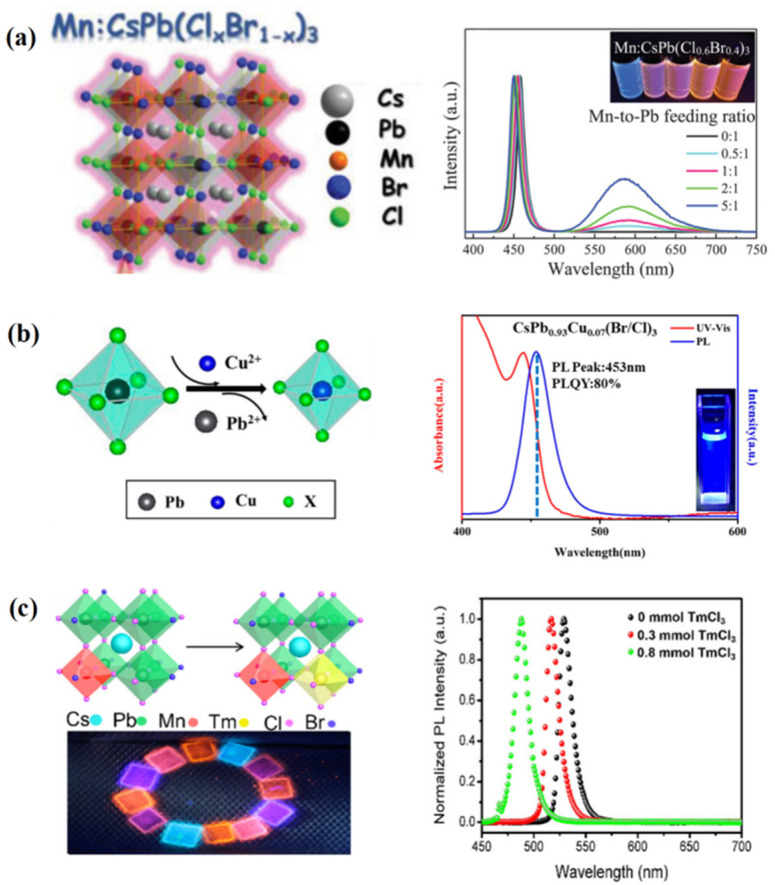
(**a**) Mn^2+^ doping. Reproduced with permission from [71]. Copyright 2019, Wiley. (**b**) Cu^2+^ doping. Adapted with permission from [67]. Copyright 2019, American Chemical Society. (**c**) Tm^3+^ doping. Adapted with permission from [66]. Copyright 2019, American Chemical Society.

The B-site contributes greatly to the conduction band of QDs, which mainly affects their photoelectric properties. The strategy of B-site doping can reduce the lead content to a certain extent, which is very important for PQDs. Commonly used doped ions include Eu^3+^ [61], Bi^3+^ [64,65], Tm^3+^ [66], Cu^2+^ [67], Zn^2+^ [68], Fe^2+^ [69,70], Mn^2+^ [64,66,71] (Figure 5a), CO^2+^ [72], etc. In 2018, Liu et al. [61] doped with Eu^3+^ to increase PLQY from 10.3% to 31.2%. In 2019, Bi et al. [67] doped Cu^2+^ ions into PQDs to improve its thermal stability and optical properties. The prepared PQDs showed bright blue photoluminescence (PL) at 450~460 nm, with a quantum yield of more than 80% and excellent thermal stability (Figure 5b). In 2019, Hu et al. [69] doped with Fe^2+^ to improve the size uniformity and PLQY of CsPbCl_3_ QDs, which also could reduce the defect recombination and non-radiation recombination of PQDs and prolong the average fluorescence lifetime. In 2021, Gualdrón-Reyes et al. [73] used 7% Sr instead of Pb to achieve FAPb_1−x_Sr_x_I_3_ PQDs with 100% PLQY and high stability for 8 months under a relative humidity of 40~50%, and T_80_ = 6.5 months. These are among the highest values reported for halide PQDs under air ambient conditions until now. FAPb_0.93_Sr_0.07_I_3_ PQDs also showed light brightening enhancement under UV irradiation for 12 h and the PLQY recovered to 100% in 15 days after synthesis. Among many doped ions, Mn^2+^ was the most concerned. The doping of Mn^2+^ could bring new energy levels and match the energy band structure of CsPbX_3_, which can realize the band edge luminescence and doped luminescence of CsPbX_3_ QDs at the same time [71]. The doped luminescence fluorescence lifetime of Mn^2+^ was up to milliseconds, which was an important basis for display applications.

Compared with IPQDs, organic perovskite prepared through a solution process could well be integrated into the silicon-based wafer. Organic molecules in perovskite materials show high polarizability, but larger molecules are required in the process of high polarization, and need to increase the hole space of perovskite. Given this, (DCl)(NH_4_)(BF_4_)_3_ was synthesized by introducing a BF_4_^−^ group at the X-site, showing a linear photoelectric coefficient of 20 pm V^−1^, which was 10 times higher than that of metal halide perovskite [74]. The photoelectric response of organic perovskite materials was close to LiNbO_3_ (Reff ≈ 30 pm V^−1^).

Multiple ion doping is another effective way to adjust exciton dynamics and realize white light emission. CsPbCl_3_ QDs doped with Bi^3+^ and Mn^2+^ were synthesized by Shao et al. [64]. By strictly controlling the concentration of doped ions, the white light emission was composed of blue QD band edge luminescence, green Bi^3+^ doped luminescence, and red Mn^2+^ doped luminescence. It was the first time to achieve the white light emission with a single component; at the same time, the correlated color temperature (CCT) could be adjusted from 4250 K to 19,000 K. In 2019, Luo et al. [66] synthesized doped CsPbBr_2.2_Cl_0.8_ QDs with Tm^3+^ and Mn^2+^, and introduced the ^1^G_4_ level of Tm between the conduction band of the QD and the ^4^T_1_ level of Mn, which effectively promoted the energy transfer of exciton from the QD body to the Mn^2+^ doped level (Figure 5c). Single component white-light QDs were obtained with a PLQY of 54%. The problem that excessive Mn^2+^ led to a sharp decline in PLQY was solved. Doping Tm^3+^ significantly improved the air stability and thermal stability of QDs, which was instructive for obtaining single-component white-light QDs with high PLQY and further display application.

Table 2 summarizes the luminescence properties of PQDs realized by different types of ion doping, including PL, FWHM, PLQY, and the lifetime after doping. 

### 4.2. Ligand Modification of PQDs

As mentioned above, OA and OAm are mostly used as ligand materials during the synthesis of PQDs. These long-chain organic ligands are attached to the surface of PQDs and have a strong protective effect on PQDs. However, OA and OAm can easily fall off after proton exchange, resulting in the degradation of the optical properties and color stability of PQDs [75]. In addition, the insulation of long-chain organic ligands will hinder charge transmission, leading to the poor conductivity of the material, which limits its application in the display field. Therefore, choosing appropriate ligands to replace long-chain organic ligands is an important way to ensure the stability and promote the application of PQDs.

**Figure 6:**
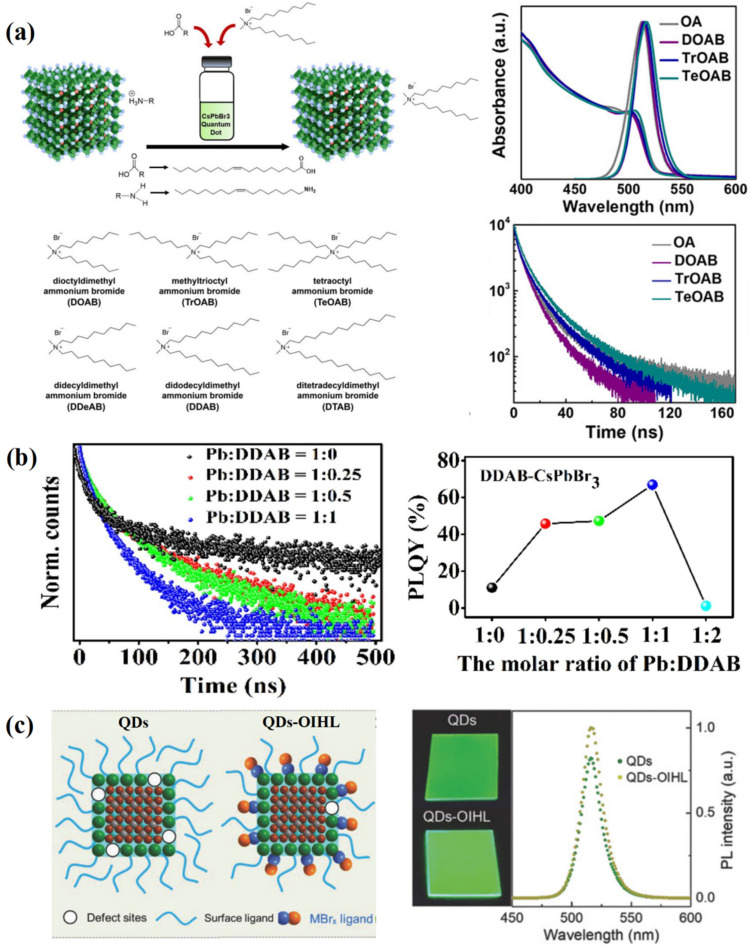
(**a**) Quaternary ammonium bromide ligand materials with different bulkiness and length. Adapted with permission from [78]. Copyright 2019, American Chemical Society. (**b**) PLQY and PL decay curves of DDAB-CsPbBr_3_ QDs with different molar ratios of Pb: DDAB. Reproduced with permission from [80]. Copyright 2021, ELSEVIER. (**c**) OIHL passivation strategy for IPQDs. Reproduced with permission from [82]. Copyright 2018, Wiley.

The volume and chain length of ligands have a significant impact on the optical properties and stability of PQDs. In 2018, Song et al. [76] utilized the synergy effect with three short ligands: tetraoctylammonium bromide (TOAB), DDAB, and octanoic acid. This effectively enhanced charge injection and transportation in QD films. The highest EQE of the prepared LED devices reached 11.6%. In 2019, SCN^−^ was used as ligands to modify the surface of CsPbX_3_ [77], which reduced Pb^2+^ surface defects and improved PLQY. The performance of LED devices prepared by modified IPQDs was improved by 25%. In the same year, Park et al. [78] found that small ligands surrounding the surface of IPQDs could effectively passivate the surface and reduce aggregation (Figure 6a). Finally, the IPQDs optimized by DDAB ligands was used to construct an efficient green LED with the current efficiency (CE) of 31.7 cd/A and the EQE of 9.7%, which is 16 times higher than the perovskite LED with traditional OAm ligands. In 2020, Huang et al. [79] synthesized DDAB-capped CsPbI_3_ QDs. The introduced DDAB could firmly be combined with the surface of PQDs and effectively passivate surface defects. The DDAB-CsPbI_3_ NCs retained PLQY > 80% for at least 60 days. In 2021, Li et al. [80] explored the influence of DDAB on the optical performance of CsPbBr_3_ QDs. They also studied DDAB-CsPbBr_3_ QDs synthesized from different molar ratios of Pb:DDAB (Figure 6b). In the same year, Yang et al. [81] fabricated CsPbBr_3_ QDs using a one-step microwave method, where α-phase poly(vinylidene fluoride) (PVDF) acted as the surface-capping ligands. The PLQY of PVDF-CsPbBr_3_ QDs reached up to 98% (much higher than pristine PQDs with OA/OAm as capping ligands). Moreover, these PQDs had excellent stability in the desired cubic phase structure and enhanced PL stability under ambient conditions.

Replacing organic ligands with inorganic materials is also an effective measure to improve the conductivity of IPQDs. In 2018, Song et al. [82] adopted an organic–inorganic hybrid ligand (OIHL) to passivate IPQDs to control its surface state and subsequently construct an efficient LED device. The inorganic ZnBr_2_ ligand could enhance the radiation recombination and carrier transport of IPQDs. In addition, it was also found that other metal bromides (MnBr_2_, GaBr_3_ and InBr_3_) could play the same role (Figure 6c). In 2020, Yang et al. [83] introduced K^+^ to partially replace organic ligands, which not only inhibited non-radiative recombination to obtain blue CsPb(Br/Cl)_3_ with the PLQY of 38.4%, but also improved the charge carrier transport performance of IPQDs. They finally obtained a stable and efficient blue perovskite LED with an EQE of 1.96%.

Compared with the direct substitution of ligands, the change in chemical bonds between ligands is also worth considering. Jang et al. [84] found that the formation of chemical bonds could effectively improve the stability of PQDs. They reported extremely stable crosslinked perovskite NPs, in which the unsaturated hydrocarbons in both the acid and base ligands of NPs were chemically crosslinked with a methacrylate-functionalized matrix, preventing decomposition of the perovskite crystals. Counterintuitively, water vapor permeating through the crosslinked matrix could chemically passivate surface defects in the NPs and reduce non-radiative recombination. Green-emitting and white-emitting flexible large-area displays were demonstrated which were stable in air and water for > 400 days. The design strategies provided a meaningful breakthrough toward the commercialization of perovskite NPs in display applications.

### 4.3. Coating of PQDs

The dynamic characteristics and low lattice energy of PQDs lead to their dissolution in almost all polar solvents, even in water. This problem continues to plague researchers until there is a strategy to completely encapsulate PQDs with inert shell materials. Compared with the ion doping and ligand modification strategies mentioned above, the coating strategy is more straightforward to improve performance by isolating as much water and oxygen as possible. So far, the silica (SiO_2_) coating method has been widely used in traditional QDs [85,86,87], such as lanthanide-doped QDs and magnetic nanocomposites. It is worth noting that SiO_2_ is an inorganic oxide with chemical stability and optical transparency within the whole visible spectrum. Coating PQDs with SiO_2_ not only retains the optical properties of luminescent materials, but also protects the materials from the dissolution by polar solvents. The silica coating for CsPbX_3_ QDs is a typical process using Stöber [88] or reverse microemulsion methods [89]. Hu et al. [90] proposed a simple and easy method to grow silica shells on CsPbX_3_ QDs in situ without using any water. During the preparation process, the amorphous SiO_2_ layer was rapidly formed on PQDs through high-temperature injection of the silica precursor tetraethyl orthosilicate (TEOS). Compared with pristine CsPbBr_3_ QDs, the performance of the prepared CsPbBr_3_/SiO_2_ composites has been significantly improved, such as luminous intensity, nonblinking properties, and optical stability.

**Figure 7:**
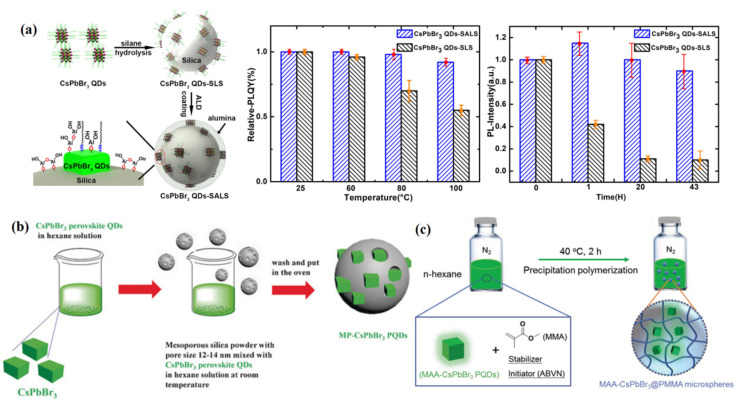
(**a**) Schematic diagram of selective surface passivation of CsPbBr_3_ QDs silicon luminescent sphere (SALS) by ALD (left); thermal stability, and photostability of as-prepared CsPbBr_3_ QDs-SLS and QDs-SALS (right). Adapted with permission from [95]. Copyright 2018, American Chemical Society. (**b**) The synthesis process of mesoporous silica green PQDs nanocomposite (MP-PQDs). Reproduced with permission from [96]. Copyright 2016, Wiley. (**c**) Illustration of the preparation process of MAA-CsPbBr_3_@PMMA microspheres. Reproduced with permission from [103]. Copyright 2019, Wiley.

In addition to the silica coating strategy, Chen et al. [91] prepared CsPbX_3_/ZnS heterostructures using a simple liquid-phase process in which QDs can be maintained in the air for 12 days. Li et al. [92] proposed a method to obtain monodisperse CsPbBr_3_/TiO_2_ core/shell NCs. The prepared monodisperse CsPbBr_3_/TiO_2_ nanocomposites have excellent water stability and PL intensity. In addition, atomic layer deposition (ALD) is an advanced method of depositing single-atom thickness films to achieve high-quality thickness control [93,94]. Xiang et al. [95] coated nanoscale alumina on the CsPbBr_3_ QDs-silica luminescent sphere via atomic layer deposition (ALD) technology to selectively passivate the surface defect sites of CsPbBr_3_ QDs (Figure 7a). The inorganic alumina coating layers can effectively reduce the ion migration and crystal deformation of CsPbBr_3_ QDs.

At present, PQDs can be coated with various mesoporous materials, such as TiO_2_, AlO_X_, SiO_2_, and so on. Wang et al. [96] mixed green CsPbBr_3_ PQDs with mesoporous silica whose pore size was approximately 12~15 nm, as shown in Figure 7b. In addition, mixing green QD-containing mesoporous silica nanocomposites with red PQDs can prevent the anion-exchange effect, improve thermal and optical stability, and finally realize on-chip LED devices with 113% NTSC. Dirin et al. [97] reported that PQDs were formed in situ assisted with mesoporous silica. High-quality PQDs were obtained by infiltrating the perovskite precursor solution into the mesoporous material and drying. Malgras et al. [98] controlled the growth of the material and found that its emission spectrum under normal environmental and light conditions remained almost unchanged.

Inorganic salts could be another choice to coat PQDs to improve their stability. Yang et al. [99] selected NaNO_3_ as a coating material for CH_3_NH_3_PbBr_3_ QDs. The precursor was dissolved in DMF solvent and then transferred to the toluene solvent. After that, CH_3_NH_3_PbBr_3_/NaNO_3_ NCs were obtained through a one-step reprecipitation procedure, and their thermal stability and photostability were greatly improved. Dirin et al. [100] used a multi-step method to obtain effective and stable PQDs through a microcarrier-assisted inorganic shell, in which PQDs were first anchored on a salt carrier and then coated with an inorganic shell through the surface-mediated reaction of the precursor. The thermostability could be significantly improved from the obtained powder. Wei et al. [101] integrated CsPbBr_3_ into CaF_2_ NPs, which greatly improved the stability to moisture, light radiation, and anion exchange.

Embedding PQDs into a polymer matrix to obtain stable QD films is one of the most popular technologies, because it does not require expensive vacuum equipment. Shen et al. [102] synthesized polymer-coated CsPbBr_3_ QDs in situ on a template. The conjugated linoleic acid (CLA) was used as a ligand to passivate the surface defects of QDs, and the CLA crosslinking was triggered under light excitation to form polymer coating and improve the stability of QDs. The FWHM of the prepared composite was narrow, and the PLQY could reach 79.16%. More importantly, the PL intensity could be maintained at 77% after soaking in water for one week due to the protection of the hydrophobic polymer layer. Cai et al. [103] reported an easy synthesis of water-resistant CsPbBr_3_ PQDs loaded poly(methyl methacrylate) (PMMA) composite microspheres (CsPbBr_3_@PMMA). Traditional OA was replaced with methacrylic acid (MAA), and the double bonds from methyl methacrylate (MMA) helped CsPbBr_3_ PQDs be polymerized into PMMA (see Figure 7c). Furthermore, a wide-color-gamut (~129%) white LED was demonstrated by combining the green-emitting CsPbBr_3_@PMMA composite microspheres and red-emitting K_2_SiF_6_: Mn^4+^ with a blue LED, which could be used as backlight for liquid crystal displays (LCD).

The glass embedding method is another feasible way to stabilize CsPbX_3_ QDs during synthesis [104]. The size of the prepared CsPbX_3_ QDs can be regulated through melt quenching and subsequent thermal treatment, and finally surrounded by a solid glass matrix. These stable CsPbX_3_ NCs could be easily integrated into display or lighting applications. Hou et al. [105] prepared a composite material based on a metal-organic framework (MOF), zeolitic imidazolate framework (ZIF) glass, and all-inorganic perovskite. It was proved that the interface development process during liquid-phase sintering played an important role in stabilizing the optically active phase of CsPbX_3_. The rigid, hydrophobic agZIF-62 provided protection for CsPbI_3_, leading to stable PL emission for (CsPbI_3_)_0.25_ (agZIF-62)_0.75_ after extended (~20 h) sonication in various nonpolar, polar protic, and polar aprotic organic solvents. In addition, the composite also exhibited stability against 10,000 h immersion in water, storage under ambient conditions for 650 days, mild heating, and continuous laser excitation for >5000 s.

Table 3 summarizes the coating of PQDs realized with different encapsulation materials and methods.

## 5. Progress of PQDs in Displays

**Figure 8:**
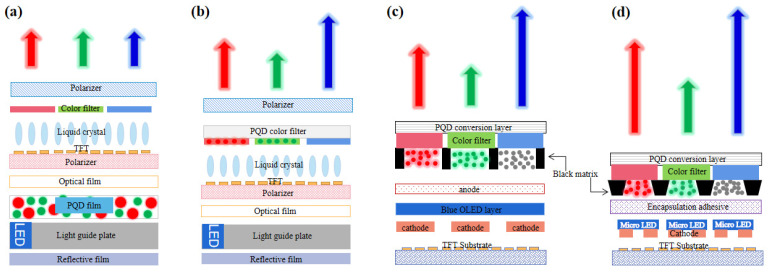
Photoluminescence display application of PQDs: (**a**) QD backlight; (**b**) QDCF; (**c**) full-color OLED with QDCCLs; (**d**) full-color micro-LED with QDCCLs.

PQDs has been used for LCD backlights as a semiconductor photoluminescent material. The basic principle is similar to traditional QDs based on their PL characteristics [106]. The white backlight source could be obtained through PQD color conversion and blue LED excitation, and then full-color display can be realized by the color filters inside the LCD panel. PQDs can also be used in a QD color filter (QDCF), which is directly collocated with blue light sources to realize a full-color display. In addition, using green and red QD color conversion layers (QDCCLs) integrated into the display panel is another color realization strategy for self-emissive displays [107,108,109,110]. The green and red emission can be obtained with the excitation of the blue OLED or blue LED [111,112]. The display applications based on the PL characteristic of PQDs are shown in Figure 8 for clear comparison. Electrically driven QLED based on electroluminescent (EL) properties will be discussed further.

### 5.1. Display Applications Based on PQD Photoluminescence

#### 5.1.1. PQD Backlight

The LCD’s color gamut is mainly determined by the backlight and color filter, and the FWHM of the light-emitting material determines the color coordinates of the backlight. In traditional white LED backlights, the spectrum is composed of blue (~450 nm) and yellow light (~580 nm) with a wide spectrum. However, in QD-based backlight, the spectrum is composed of three narrow emission peaks of red, green, and blue (RGB).

Compared with most traditional luminous materials, PQDs have the characteristics of higher efficiency and narrower FWHM, which would become a representative material in displays in the future. Taking the edge-lit LED backlight as an example, the PQD’s backlight technology can be divided into four types according to the encapsulation methods.

**Figure 9:**
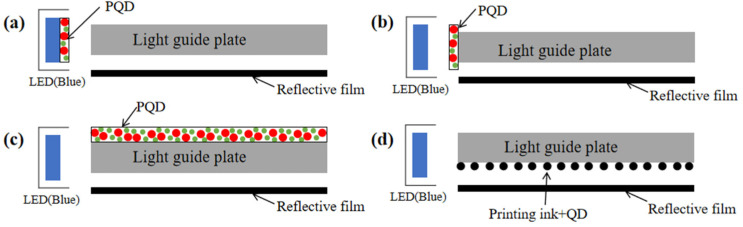
Schematic diagram of PQD backlight structures. (**a**) On chip; (**b**) on edge; (**c**) on surface; (**d**) in dot.

**(1) On chip (chip direct contact).** As shown in Figure 9a, PQDs replaces the traditional phosphor materials and are directly encapsulated on the blue LED chips to obtain a white light source [113]. Although this structure has the merits of easy realization and low cost, it requires a highly stable performance of PQDs to ensure efficient luminescence. This is mainly because the PQDs have to be directly in contact with the LED chip. A normally working LED chip will emit dramatically increased heat. The above PQDs have to maintain stability at a temperature even higher than 150℃, which is still a huge challenge to PQDs currently.

**(2) On edge (glass tube encapsulation).** As shown in Figure 9b, PQDs are encapsulated into a long glass tube, and placed on the side of a light guide plate with a blue LED light bar. In this structure, PQDs are not in direct contact with a blue LED chip, so the influence of thermal radiation and light radiation from LED chips can be greatly reduced. It has been used for display backlights in 2013, which was called “QD optical tube technology” developed by QD vision in the United States [114]. However, the existing QD glass tube encapsulation technology has the problems of low luminous efficiency (LE) and unfavorable assembly operation, which limits its large-scale application and development. In addition, it is still uncertain whether the environmental isolation level of ordinary glass can meet the need of PQDs.

**(3) On surface (optical film or plate integration).** As shown in Figure 9c, the PQD film can be placed directly above the light guide plate, and the blue LED is placed on the side of the backlight module. The blue light emitted by the LEDs is shaped into a uniform blue surface light through the joint action of the light guide plate and the bottom reflection film. The blue surface light source then excites PQD film to form a white backlight source. Another on-surface configuration may be the PQD diffuser plate, which should be used for direct-lit mini-LED backlights [115,116]. Because PQDs are far enough from blue LED, they are hardly affected by the thermal radiation and light radiation of the LED chip. Meanwhile, the multi-layer structure also provides a potential to isolate the PQD layer from the environment. The key problem is how to balance the ratio of the three primary colors for a white backlight with a higher stability and a wider color gamut.

**(4) In dot (micro-structure integration).** As shown in Figure 9d, the micro-structure is fabricated by a screen printing or an inkjet printing process which contains the mixture of red/green QDs and ink, then the mixed QD slurry is printed on the bottom surface of the light guide plate for light mixing and diffusion [94,106,117]. The red and green emissions are converted by red and green QDs under the excitation of the edge-lit blue LED. These micro-structural arrays can be optimized for better uniformity and extraction efficiency [118]. Uniform white light could be achieved by adjusting the proportion of the red and green QDs in the mixture. The current challenge is the ink configuration and environmental exposure.

These photoluminescent PQD devices face their own respective problems. The “on chip” solution is more straightforward and easier to prepare, so it has become the primary choice for researchers. The “on chip” backlight can achieve good white light with color mixing in a limited encapsulation space. In 2015, Zhang and co-workers [25] developed white down-converted (DC) LEDs with a wide color gamut by using HPQDs MAPbBr_3_ (green-emitting) and rare-earth K_2_SiF_6_:Mn^4+^ phosphors (red-emitting) as color down-converters. This device had CIE coordinates of (0.33, 0.27), 130% NTSC, and an LE of 48 lm/W at 4.9 mA, which could well match the need of display backlights. To further improve the stability, Wang et al. [96] reported an “on chip” structure by introducing mesoporous silica composites. It should be noted that the prepared white-light device demonstrated 113% NTSC after passing through a color filter without the anion exchange issue. In 2022, Wang et al. further showed that the PLQY improvement of full-visible-spectrum IPQDs could be successfully attained by a renewable and low-cost anion exchange resin. The PLQY of three-primary-color IPQDs could be dramatically improved to 93.69%, 89.99%, and 65.03%. Meanwhile, the prepared LED by “On chip” solution provides high brightness and a wide color gamut simultaneously [115]. Although the important display parameters, including the color restoration and stability improvement, have been the focus of attention, the potential of different PQD photoluminescent devices still deserves to be further explored.

#### 5.1.2. PQD Color Conversion Layer

Color definition of LCD is realized through the backlight and color filter in the liquid crystal panel. In addition to backlight, PQDs can also be introduced into a color filter as CCLs, as shown in Figure 8b–d. By employing blue OLED or micro-LED as the excitation source, almost all blue light is converted into desired red or green light by PQDCCLs to realize full-color displays. The blue light should be absorbed as much as possible to reduce eye damage. Therefore, by using PQDs as CCLs, the first consideration is how to maintain the high PLQY while ensuring stability.

Yang et al. [119] compounded PQDs and thermoplastic elastomer into a stretchable and self-healing filter film. Furthermore, they fabricated and sequentially stacked green (MAPbBr_3_) and red (CsPbBr_0.6_I_2.4_) composite films, which can be excited by a blue LED to realize white light. It is worth mentioning that PQDs’ polymer films with humidity self-healing properties have been further discussed by Cai et al. to support the potential use of polymer conversion films in displays [120]. Yin et al. [121] developed vacuum drying perovskite film preparation technology, and successfully prepared micron-level perovskite films with uniform thickness. The perovskite film with a thickness of 3.8 µm was excited by using 463 nm blue OLED or micro-LED. The brightness of the generated green emission could reach 200 cd/m^2^ when the brightness of the excitation source was at 1000 cd/m^2^. In addition, only 2% brightness attenuation was observed after 18 days of exposure to the environment. In 2020, Hu et al. [122] used inkjet printing and UV-induced polymerization to obtain micron-thick QD films with uniform surface morphology. By combining QD films with blue OLED or micro-LED displays, green displays were successfully realized via color conversion. Due to the applicability of wet preparation, replacing traditional QDs with PQDs is also feasible, which can support the development of PQDCCLs.

Photoluminescence displays or backlights occupied an important position for the potential application of PQDs. The studies on traditional QD photoluminescence application are increasingly mature these days. The opportunity of PQD’s deeper permeation into displays is the way to replace traditional color conversion material and find effective ways to enhance stability.

### 5.2. Display Applications Based on PQD Electroluminescence

In terms of PQD electroluminescence, quantum-dot light-emitting diodes (QLED) have aroused worldwide concern for decades. They are naturally compatible with attractive flexible or bendable display devices. The booming development of QLED requires the exploration of more efficient emitters, such as PQDs. The characteristics, including high radiative recombination, high defect tolerance, and excellent optical properties, have allowed them to be a rising star as QLED emitters. Numerous studies have been devoted to perovskite QLED (PeQLED) to enhance performance through composition design, surface engineering, and device structure modification [82,123,124].

The first PeQLED was fabricated with MAPbBr_3_ QDs in 2014 by Schmidt et al. [30]. The demonstrated luminance of this device was extremely low, which was even lower than 1 cd/m^2^. Later on, Huang et al. [125] employed size-tunable MAPbBr_3_ QDs to fabricate QLED, which exhibited improved performance with a maximum brightness of 2503 cd/m^2^, current efficiency (CE) of 4.5 cd/A, power efficiency (PE) of 3.5 lm/W, and 1.1% EQE. Xing et al. [126] adopted the device structure of ITO/PEDOT: PSS/MAPbX_3_ QDs/TPBi/Cs_2_CO_3_/Al and obtained superior performance with CE of 11.49 cd/A, PE of 7.84 lm/W, and 3.8% EQE. Yan et al. [123] presented an efficient QLED based on MAPbBr_3_ QDs by achieving charge balance and suppressing the Auger recombination under a low driving voltage. This device showed a maximum luminance of 43,440 cd/m^2^, a PE of 30.3 lm/W, and 12.9% EQE.

**Figure 10:**
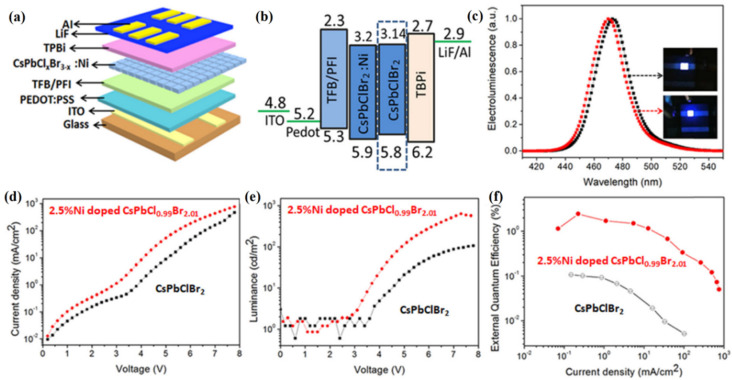
(**a**,**b**) Schematic diagram and band level of the PeQLED based on 2.5% Ni^2+^ ion-doped CsPbCl_0.99_Br_2.01_ QDs. (**c**) The EL spectra and photos of the PeQLED based on CsPbClBr_2_ QDs (black) and 2.5% Ni^2+^—doped CsPbCl_0.99_Br_2.01_ QDs (red), respectively. (**d**–**f**) Current density, luminance, and EQE of the corresponding devices. Adapted with permission from [130]. Copyright 2020, American Chemical Society.

As an effective strategy, mixing cations have been employed to construct high-efficiency PeQLED. Cho et al. [127] first reported the synthesis of MA_1−x_Cs_x_PbBr_3_ QDs and the corresponding MA_0.7_Cs_0.3_PbBr_3_-based QLED, which had a maximum luminance of 24,510 cd/m^2^, a CE of 4.1 cd/A, and 1.3% EQE. Zhang [128] exploited the mixed cation FA_0.8_Cs_0.2_PbBr_3_ to fabricate QLED, which achieved a high luminance of 55,005 cd/m^2^, a CE of 10.09 cd/A, and 2.80% EQE. Red FA_0.87_Cs_0.13_PbI_3_ NCs with γ-butyrolactone was utilized as a solvent to realize a QLED with a maximum luminance of 218 cd/m^2^ and a peak EQE of 15.8% in 2019 [129]. Pan et al. [130] incorporated Ni^2+^ ions into CsPbCl_x_Br_3__−x_ QDs by a supersaturated recrystallization synthetic method and modulated the Cl/Br element ratios to obtain an efficient blue PeQLED. The corresponding device presented a maximum luminance of 612 cd/m^2^ and 2.4% EQE (Figure 10).

**Figure 11:**
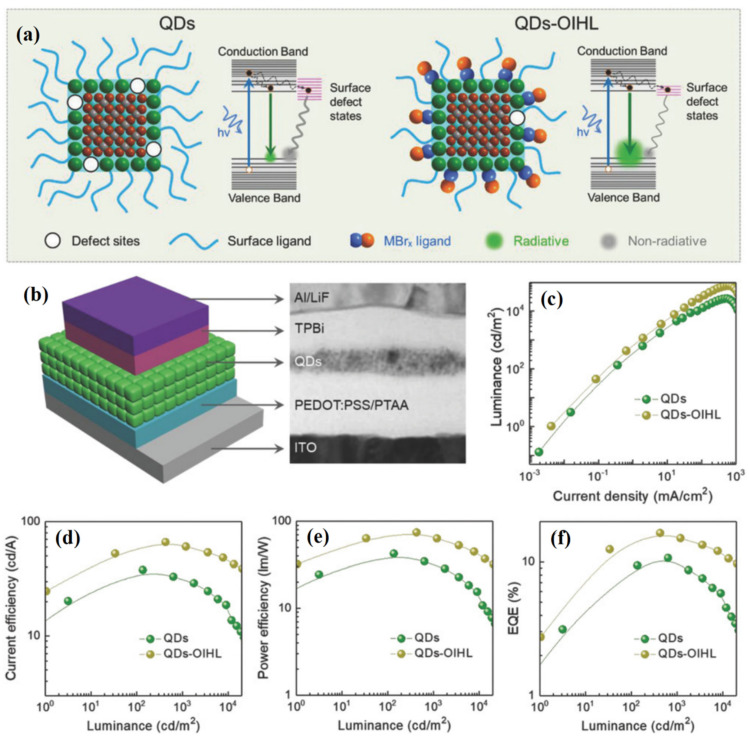
(**a**) Schematic of radiative/nonradiative recombination of PQDs with and without OIHL. (**b**) Illustration of multilayer PeQLED device and cross-sectional TEM image. (**c**–**f**) Luminance, CE, PE, and EQE of the devices. Reproduced with permission from [82]. Copyright 2018, Wiley.

Actually, surface ligand engineering plays an important role in the performance enhancement of PeQLED. Although surface capping ligands could remove surface trap states to improve the stability of PQDs, they also form an insulating film to hinder the charge injection and transport inside the light-emitting layer of PeQLED. Therefore, appropriate surface ligand modification benefits the performance enhancement of PeQLED. Lee et al. [131] utilized a short amine ligand to enhance the efficiency of FAPbBr_3_-based PeQLED. The reported FAPbBr_3__−n_-butylamine-based PeQLED had a high performance with CE of 9.16 cd/A, a PE of 6.4 lm/W, and 2.5% EQE. Han [124] exploited 3,3-diphenylpropylamine bromide (DPPA-Br) as surface capping ligands to fabricate uniform FAPbBr_3_ QDs. The resulting PeQLED demonstrated an excellent performance with a maximum luminance of 13,970 cd/m^2^, a CE of 66.3 cd/A, and 16.3% EQE. Song et al. [82] explored an organic-inorganic hybrid ligand to passivate the surface trap state and enhance the carrier injection of PQDs, including the use of ZnBr_2_, MnBr_2_, GaBr_3_, and InBr_3_. As a result, the ZnBr_2_-passivated PeQLED showed superior performance with a maximum luminance of 76,940 cd/m^2^, a CE of 66.7 cd/A, a PE of 65.9 lm/W, and 16.48% EQE, while the MnBr_2_-passivated PeQLED presented a maximum luminance of 100,080 cd/m^2^, a CE of 60.6 cd/A, and 15.6% EQE (Figure 11). In 2021, Li et al. [132] used FABr to compensate the surface Bromine vacancy of inorganic cesium lead halide PQDs. The corresponding PeQLED with FABr-treated CsPbBr_3_ QDs (green-emitting) achieved a promising EQE of 7.94% and a luminance of 14,790 cd/m^2^, which was much higher than those of the pristine (1.78%, 4640 cd/m^2^) and the DDAB-treated samples (3.91%, 167 cd/m^2^). This methodology is also suitable for the surface defect passivation of blue-emitting CsPbBr_1.3_Cl_1.7_ and red-emitting CsPbBrI_2_ QDs. The results clearly highlighted that heterogeneous post-passivation could boost the PL and EL performance of inorganic cesium lead halide PQDs simultaneously.

Surface ligand passivation can improve the PLQY of QD, but their insulating nature impedes the charge injection in a QD film and further negatively impacts the PeQLED’s performance. Hence, appropriate ligand choice and effective ligand exchange play important roles in building a high-efficiency PeQLED. Moreover, optimizing device inter-layer structures with matched energy levels to increase radiative recombination efficiency is another major method in the achievement of efficient PeQLED. Recently, Khan et al. [133] optimized the device architecture and energy level matching of CsPbBr_3_-based PeQLED through the introduction of Li-doped TiO_2_ nanoparticles as an electron transport layer (ETL). Compared with the untreated device (3 V turn-on voltage and 5.6 cd/A CE), the resulting device exhibited 2V turn-on voltage and an enhanced CE of 15.2 cd/A with the Li-doped TiO_2_ ETL.

Other effective measures are proposed to improve PeQLED. Dong et al. [134] reported PQDs resurfacing to achieve a bipolar shell consisting of an inner anion shell, and an outer shell comprised of cations and polar solvent molecules. The outer shell was electrostatically adsorbed to the negatively charged inner shell. This approach produced strongly confined PQD solids with improved carrier mobility (≥0.01 cm^2^ V^−1^ s^−1^) and reduced trap density. Efficient blue and green PeLEDs can be fabricated by exploiting CsPbBr_3_ QDs with reduced trap density and improved mobility. Tsai et al. [135] demonstrated that perovskite NCs stabilized in the MOF thin films could maintain decent PL and EL against continuous ultraviolet irradiation, heat, and electrical stress. Bright and stable LEDs were demonstrated with a maximum EQE of over 15% and a high brightness of over 105 cd/m^2^ after stabilization. During PeLED operation, the nanocrystals can be well preserved, free of ion migration or crystal merging through protection by the MOF matrix, leading to a stable performance over 50 h.

**Figure 12:**
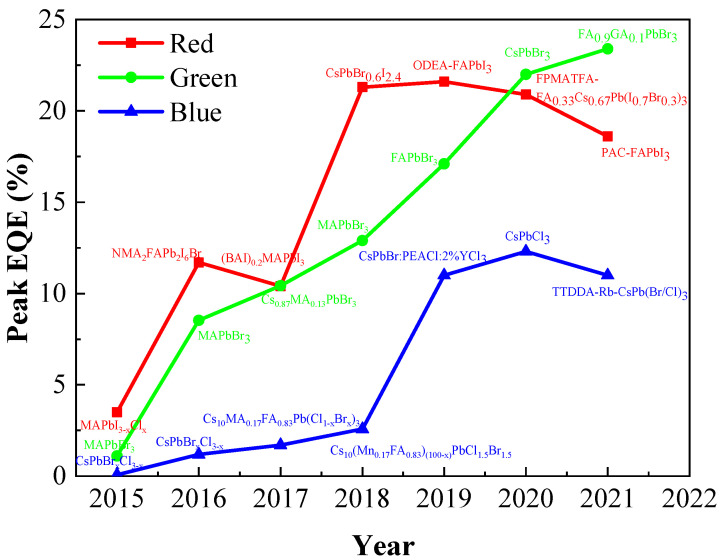
Evolution curve of the peak EQE of perovskite QLED.

Figure 12 summarizes the development of EQE of full-color PeQLED devices. Up to now, the maximum EQE values of red, green, and blue QLED have reached 21.6%, 23.4%, and 12.3%, respectively, which has increased by ~20% since 2015. However, so far, the PeQLED’s lifetime is still far lower than the commercial standard of 10,000 h. The main obstacle to the commercialization of PeQLED is its instability in the environment. Consequently, improving the performance and stability of PQDs is still the main goal for future development.

## 6. Conclusions

In this paper, we summarize the synthesis methods of PQDs and analyze the effects of ion doping, ligand modification, and coating engineering on PQDs. Furthermore, the applications of PQDs in photoluminescence and electroluminescence displays are described in detail. It is believed that the following aspects require more attention as regards the display future: (1) large-area and pixelated preparation of PQDs with excellent homogeneity; (2) use of ligand modification, ion doping, or coating strategies to synthetically improve the environmental stability of PQD while retaining high PLQY; (3) development of physical and chemical properties to realize more diverse PQD display devices, such as flexible and bendable displays; (4) lead-free PQDs with excellent luminescence efficiency and high environmental friendliness to meet the needs of sustainable development.

## Figures and Tables

**Table 1:** The existing synthesis methods of PQDs.

Methods	Principle	Results	Drawbacks	Reference
Hot injection	High temperature	High yield, good properties, suitable for ion doping and ligand modification, widely used	Complex process	[26]
Anion exchange	Doping	Full-spectrum luminescence, easy X-position doping	Multi-step process	[47]
Room-temperature reprecipitation	Different solubility	Easy operation, high repeatability, suitable for ligand modification	Uneven size	[48]
Ultrasonic method	Ultrasonic treatment	Easy operation, suitable for ligand modification	High cost	[49]
Microwave-assisted synthesis	Microwave treatment	Easy operation, high repeatability, suitable for ligand modification	High cost	[50]
Solvothermal synthesis	Mixed high temperature	Easy to synthesize, controllable morphology	System temperature unevenness, not suitable for ion doping and ligand modification	[51]
Mechanochemical synthesis	Mixed grinding	High yield, easy to synthesize	Not applicable to ligand modification	[52]
Wet ball milling	Mixed grinding	Easy to synthesize	Low synthetic efficiency	[53]
Dry ball milling	Mixed grinding, solvent-free	Fast, high synthetic purity	Easy to generate surface defects	[54]
Chemical vapor deposition	Chemical reaction, deposition	Excellent performance	Large size, precise equipment	[55]
Microfluidic platform	Carrier spacing reaction	Automatic, homogeneity	Immature	[57]

**Table 2:** Luminescence properties of different ion-doped PQDs.

Doping	Excitation (nm)	PL (nm)	FWHM (nm)	PLQY (%)	τ (ns)	Stability	Advantages	Reference
		**A—site doping**						
BA^+^	−	−	−	49.44	24.58	Stable (50 days, 80% RH)	Reduced dimensionality	[59]
K^+^	365	408	12.7	10.3	13.6	−	Greatly improved PLQY	[61]
Rb^+^	365	505–515	18–20	93	5.32	30% (100 °C, 24 h)	Increased exciton binding energy	[63]
		**B—site doping**						
Eu^3+^	365	408	11.3	31.2	15.24	−	Greatly improved PLQY	[61]
Bi^3+^	365	420–520	−	52	9.5	70% (30 days, air)	Lead-free PQDs	[64]
Tm^3+^	365	−	−	54	4.8–5	Stable (80 °C, 24 h)	Introduction of new energy level	[66]
Cu^2+^	365	450–460	15–26	>80%	2.3–5	90% (30 days, 60% RH, 25 ℃)	Eliminating halide vacancies	[67]
Zn^2+^	365/380	395–550	47	79.05	−	63.77% PLQY (50 days, air)	Lead-free PQDs	[68]
Fe^2+^	−	401–403	13.8–14.6	6.2	14.6	−	Size homogeneity improvement	[69]
Mn^2+^	365	−	−	65	−	−	Toxic ions reduction and PLQY improvement	[71]
Co^2+^	365	516	18–20	89	17.93	90% (50 days)	Defect passivation	[72]
Sr	−	589,583,530	−	100	−	Stable for 8 months (40–50% RH, 6.5 months)	Defect passivation	[73]
		**X—site doping**						
BF_4_^−^	−	515	−	−	−	−	Increased hole space of perovskite	[74]
		**Multiple ion doping**						
Bi^3+^, Mn^2+^	365	420–520	−	52	9.5	70% (30 days, air)	Wide range of CCT	[64]
Tm^3+^, Mn^2+^	365	−	−	54	4.8–5	Stable (80 °C, 24 h)	Promotion of exciton energy transfer	[66]

**Table 3:** Luminescence properties of PQDs realized with different packaging materials and methods.

Wrapping	Excitation (nm)	PL (nm)	FWHM (nm)	PLQY(%)	τ (ns)	Stability	Advantages	References
CsPbBr_3_/SiO_2_	350	533	18	−	48.3	73.8% (75% RH, air, 12 h); 36.4% (60 °C, 15 h)	Anion exchange prevention and stability improvement	[90]
CsPbX_3_/ZnS	365	−	−	70	−	−	More stable, tunable	[91]
CsPbBr_3_/TiO_2_	405	518	32	−	2.1	Stable for 12 weeks (water); ≈75% (UV, 24 h)	Suppress anion exchange and photodegradation	[92]
CsPbBr_3_/Al_2_O_3_	365	516	23	65	36.57	PL stable (96 h, water); 80% (450 nm, 200 mW/cm^2^, 40 h)	Defect passivation	[95]
CsPbBr_3_/Mesoporous silica	365	457–698	13–35	−	−	80% (365 nm, 6 W UV, 96 h)	Prevent ion exchange and increase stability	[96]
MAPbBr_3_/NaNO_3_	365	525–526	24	42	155.5	30% (100 °C, 5 h); 80% (365 nm/6 W UV, 14 h)	Improved stability	[99]
CsPbBr_3_@SiO2/Poly-CLA	365	511	20	79.16	218.11	77% (water, 1 week)	Improved stability	[102]
CsPbBr_3_@PMMA	395	514	26	32.8	122.2	91% (water, 7 days); stable (water, 1 month)	Improved water resistance and storage stability	[103]
CsPbI_3_/ZIF glass	−	−	42	>65%	17.6 (average)	80% (water, 10,000 h) no CsPbI3 phases change (air condition) active phase preserved (after 77 K) Over 80% (after 100 °C in air or 80 °C in air for 1000 cycles) 90% (57 mW/cm^2^ over 5000 s)	Improved stability	[105]

## Data Availability

Not applicable.
